# Aerosolized delivery of ESKAPE pathogens for murine pneumonia models

**DOI:** 10.1038/s41598-024-52958-9

**Published:** 2024-01-31

**Authors:** Katharina Rox, Eva Medina

**Affiliations:** 1 Department of Chemical Biology, Helmholtz Centre for Infection Research (HZI), Inhoffenstraße 7, 38124 Braunschweig, Germany; 2 German Center for Infection Research (DZIF), Partner Site Hannover-Braunschweig, 38124 Braunschweig, Germany; 3 Infection Immunology Group, Helmholtz Centre for Infection Research (HZI), Inhoffenstraße 7, 38124 Braunschweig, Germany

**Keywords:** Animal disease models, Pharmacodynamics, Experimental models of disease

## Abstract

Murine pneumonia models for ESKAPE pathogens serve to evaluate novel antibacterials or to investigate immunological responses. The majority of published models uses intranasal or to a limited extent the intratracheal instillation to challenge animals. In this study, we propose the aerosol delivery of pathogens using a nebulizer. Aerosol delivery typically results in homogeneous distribution of the inoculum in the lungs because of lower particle size. This is of particular importance when compounds are assessed for their pharmacokinetic and pharmacodynamic (PK/PD) relationships as it allows to conduct several analysis with the same sample material. Moreover, aerosol delivery has the advantage that it mimics the ‘natural route’ of respiratory infection. In this short and concise study, we show that aerosol delivery of pathogens resulted in a sustained bacterial burden in the neutropenic lung infection model for five pathogens tested, whereas it gave a similar result in immunocompetent mice for three out of five pathogens. Moreover, a substantial bacterial burden in the lungs was already achieved 2 h post inhalation. Hence, this study constitutes a viable alternative for intranasal administration and a refinement of murine pneumonia models for PK/PD assessments of novel antibacterial compounds allowing to study multiple readouts with the same sample material.

## Introduction

There is a high quest for novel antibacterials, especially against the so-called ESKAPE pathogens (*Enterococcus faecium*, *Staphylococcus aureus*, *Klebsiella pneumoniae*, *Acinetobacter baumannii*, *Pseudomonas aeruginosa*, and *Enterobacter* spp), as the rise in multidrug-resistance poses a true public health threat whereas at the same time the antibacterial development pipeline runs dry^1–3^. This still holds true despite the fact that recently, new antibacterials, including those with new modes of action like cefiderocol^4,5^, have reached the market or are under late-stage clinical evaluation^6^. Neutropenic as well as acute lung infection models are common experimental systems to provide a proof-of-concept and to explore PK/PD relationships of novel antibacterials^7^. Most frequently, the infection is introduced using an intranasal instillation^8,9^. Additional techniques consist of oropharyngeal or intratracheal instillation^10,11^. Nevertheless, all aforementioned techniques have several limitations: (1) The volume of the inoculum is restricted and narrow. For intranasal administration (depending on the country) only 30 to 50 µl are permitted to be used on one nostril; similarly, oropharyngeal as well as the intratracheal administration allow only 50–70 µl for instillation. (2) The success of the infection is highly dependent on the experimenter, as that person needs to assure that equal volumes are introduced^9^. (3) Finally, as the success of the infection depends on the breathing rate of the animal, it is possible that the infection of the lung does not occur homogeneously, but that only one lung lobe is infected^12,13^. This limits the use of different parts of the lung of the same mouse for analysis requiring different sample preparation, e.g. cfu determination procedures differ from RNA preparation and extraction procedures. It is known that aerosol delivery results in a more homogeneous distribution of particles within the lungs^14^. The aim of this study was to investigate if sustained infections were established upon aerosol infection with several pathogens of the ESKAPE group, which overcomes the aforementioned constraints of intranasal or intratracheal instillation, and to validate the models using a positive control antibacterial, i.e. levofloxacin, to demonstrate that they work in the same manner as models using intranasal or intratracheal instillation^7,15^.

## Results

### Aerosol delivery in the neutropenic pneumonia model

First, we deployed the standard neutropenic pneumonia model^7^, but instead of intranasal infection we used aerosol delivery of several ESKAPE pathogens, namely *S.* *aureus*, *K.* *pneumoniae*, *P.* *aeruginosa*, *A.* *baumannii* (two different strains) and *S.* *pneumoniae*. We chose one strain per pathogen which is well defined and frequently used for neutropenic animal models. Aerosol delivery was performed under ketamine/xylazine anesthesia which enabled that the mice were breathing slowly resulting in less inter-animal variation. Mice were held in upright position and infected individually using the Aeroneb® lab nebulizer device with a defined amount of inoculum (Supplemental video files S1–S3), which was placed carefully over mouth and nose of the individual animal in a dedicated infection cage. Afterwards, remaining droplets of the inoculum, which might have been deposited on the whiskers and/or the fur of the individual animals, were removed using a tissue before placing the anesthetized animal back to its original cage and on a warming mat until it was awake again.

**Figure 1:**
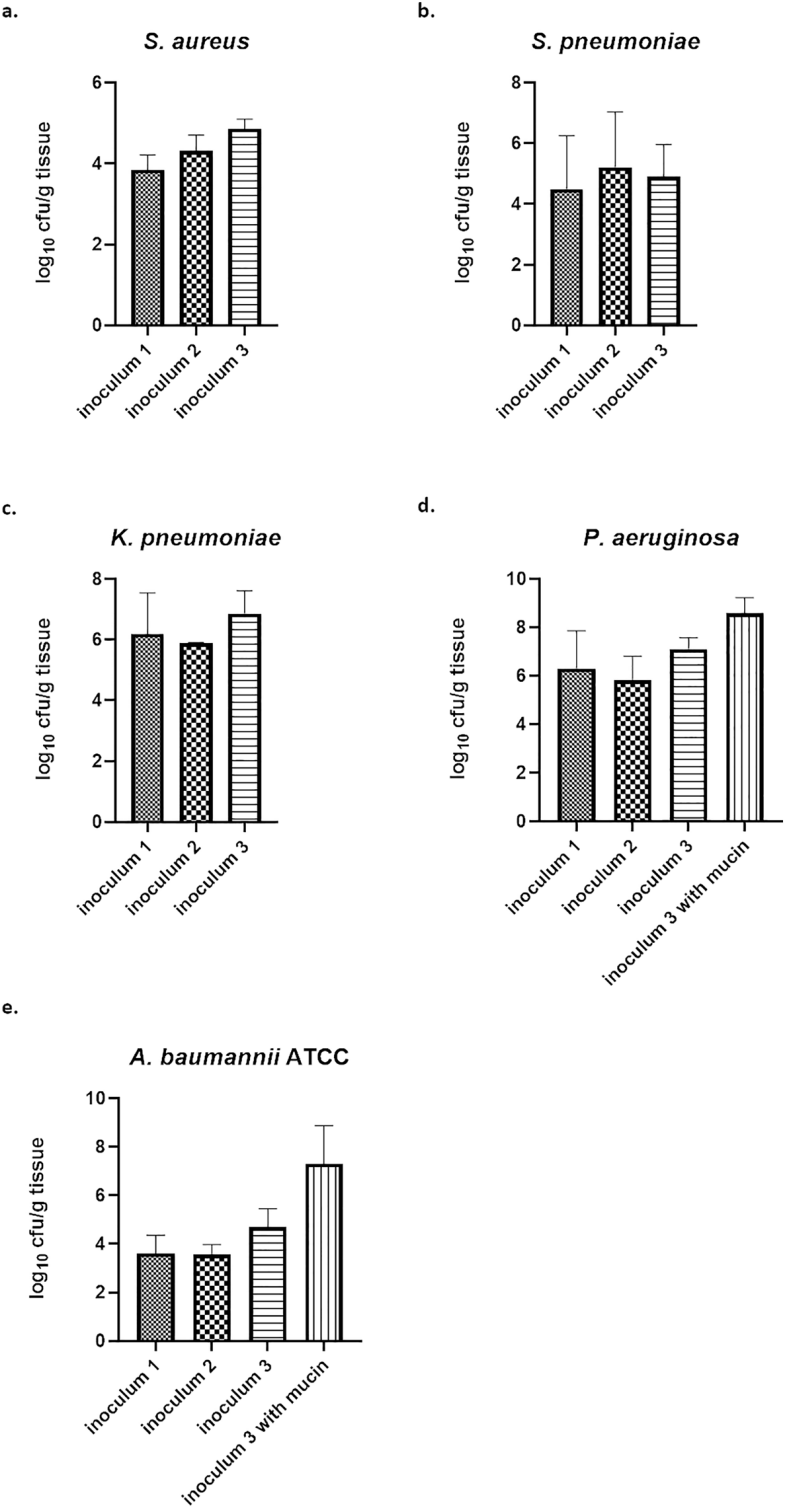
Bacterial burden after aerosol delivery of different ESKAPE pathogens in the standard neutropenic pneumonia model during inoculum titration. Bacterial burden after 24 h after aerosolized delivery of three different inocula is shown for *S.* *aureus* (**a**), *S.* *pneumoniae* (**b**), *K.* *pneumoniae* (**c**), *P.* *aeruginosa* (**d**), *A. baumannii* (**e**) in the neutropenic pneumonia model.

To determine which inoculum size was needed to achieve a sustainable burden after 24 h, three different inocula were tested for every strain (Table S1). As expected, an increase of the inoculum resulted in an increased bacterial burden for *S. aureus* (Fig. 1a). However, 2 × 10^9^ cfu/ml as inoculum only gave around 1 log_10_ unit increased burden compared to 4 × 10^8^ cfu/ml. In case of *S.* *pneumoniae*, the increase of bacterial burden at 24 h post infection was also only slightly augmenting with a higher inoculum (Fig. 1b). For *K.* *pneumoniae*, the highest bacterial burden at 24 h post infection was achieved with an inoculum dose of 2 × 10^9^ cfu/ml (Fig. 1c). For *A.* *baumannii* strain ATCC 19606 and *P.* *aeruginosa* only a moderate bacterial burden was achieved after 24 h and not much difference was observed between the three different inocula (Fig. 1d,e). However, inoculum preparation for those two strains in presence of 0.01% mucin resulted in a much higher terminal bacterial burden using the highest inoculum. Finally, with the highest inoculum as infection dose, a good terminal bacterial burden at 24 h post infection was achieved for the five different bacterial species assessed here (Fig. S1a). Whereas both Gram-positive bacterial strains of *S.* *pneumoniae* and *S.* *aureus* showed a bacterial burden of around 5 log_10_ cfu/g tissue 24 h after infection, the three Gram-negative tested strains of *K.* *pneumoniae*, *P.* *aeruginosa* and *A.* *baumannii* exhibited a bacterial burden greater than 6 log_10_ cfu/g tissue. Moreover, only a small standard deviation with respect to bacterial burden was seen for the strains of *K.* *pneumoniae*, *P.* *aeruginosa*, *S.* *aureus* and *S.* *pneumoniae*. This demonstrates that aerosolized delivery did not result in high variability (Table S2).

**Figure 2:**
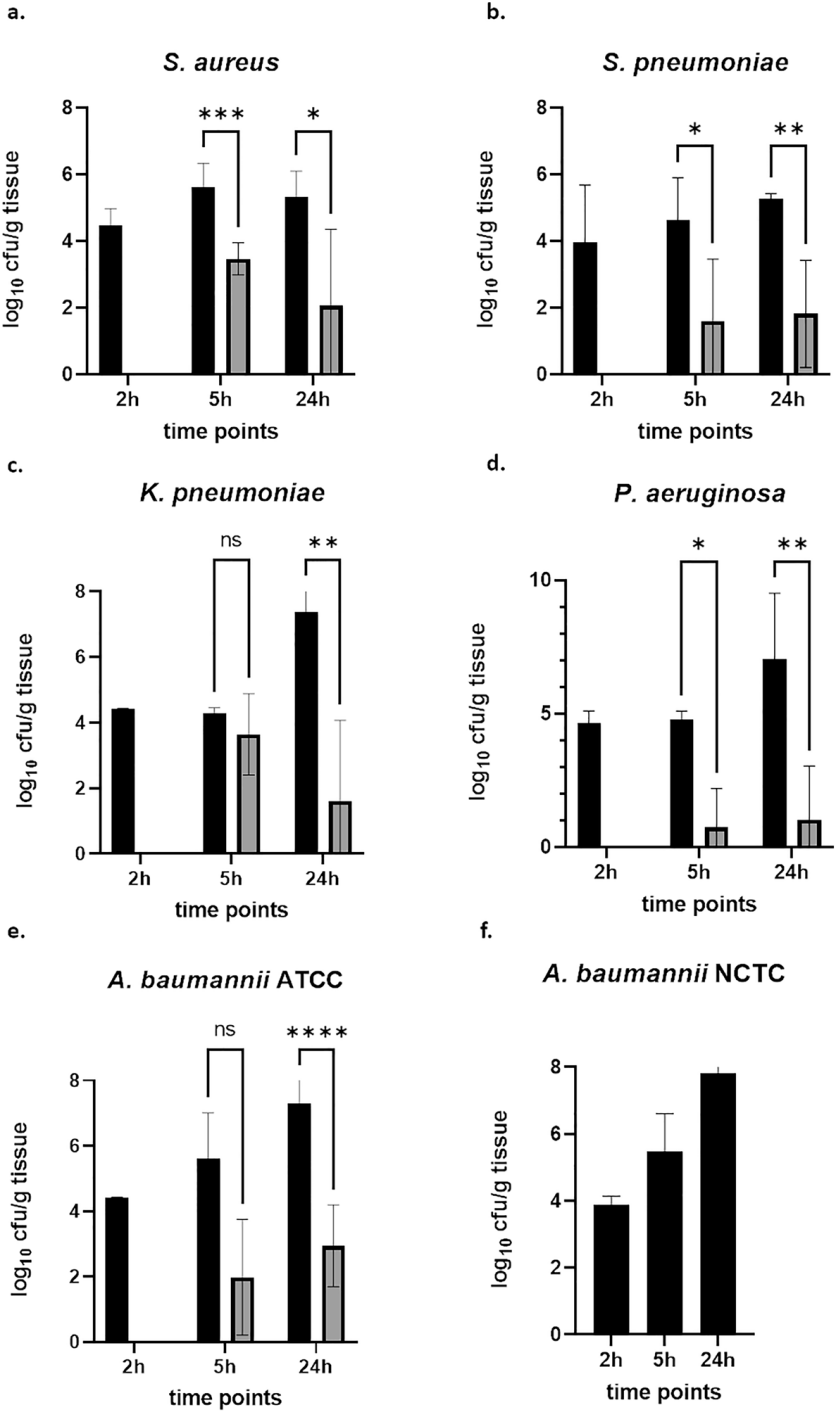
Validation of neutropenic pneumonia models with levofloxacin at different time points. Bacterial burden after 2, 5 and 24 h after aerosolized delivery is shown for *S.* *aureus* (**a**), *S.* *pneumoniae* (**b**), *K.* *pneumoniae* (**c**), *P.* *aeruginosa* (**d**), *A. baumannii* strain ATCC 19606 (**e**) and *A.* *baumannii* strain NCTC 13301 (**f**). Animals were treated with vehicle (black) or with levofloxacin (grey). Levofloxacin did reduce bacterial burden at 24 h significantly for all pathogens tested; at 5 h levofloxacin did only reduce bacterial burden for *S.* *aureus*, *S.* *pneumoniae* and *P.* *aeruginosa* significantly. *p < 0.05, **p < 0.01, ***p < 0.001, ****p < 0.0001.

Next, we wanted to validate the models using a positive control antibacterial, in our case levofloxacin. Moreover, we determined bacterial burden at 2, 5 and 24 h post infection for all five pathogens. For *S.* *aureus*, we did observe that from the two-hour time point, bacteria grew by around 1 log_10_ unit up to five hours. Furthermore, bacterial burden in the vehicle-treated groups at 5 and 24 h was nearly similar. The positive control group with levofloxacin treatment decreased bacterial burden significantly at 5 and 24 h, although a higher decrease was observed at 24 h (Fig. 2a). For *S.* *pneumoniae*, a slight increase of bacterial burden from two towards five and, finally, to 24 h was seen in the vehicle-treated group. Again, levofloxacin reduced bacterial burden significantly for both time points assessed (Fig. 2b). In case of *K.* *pneumoniae*, there was not much growth observed in the vehicle-treated group from two towards five hours. However, a substantial increase in bacterial burden was seen at 24 h. Levofloxacin did only slightly reduce bacterial burden at 5 h, whereas this reduction was significant at 24 h (Fig. 2c). Similar to *K.* *pneumoniae*, also for *P.* *aeruginosa* not much growth from two to five hours with respect to bacterial burden was observed in the vehicle-treated group, but a substantial growth was seen at 24 h. Levofloxacin did significantly reduce bacterial burden at 5 and 24 h compared to the vehicle-treated groups at the same time points (Fig. 2d). Similar to *S.* *pneumoniae*, growth from two towards five and 24 h was observed for *A.* *baumannii* strain ATCC 19606. Levofloxacin did reduce bacterial burden at 5 h, but missed significance, whereas the reduction was significant at 24 h (Fig. 2e). To get a first idea if the method might also be suited for strains of the same bacterial species, we used *A.* *baumannii* strain NCTC 13301 with the same inoculum (in presence of mucin) as established for the strain ATCC 19606 (Fig. 2f). Similar growth was observed as seen for the strain ATCC 19606 giving first hints that the aerosolized delivery of the pathogen is not limited to particular strains.

Finally, we show that the neutropenic lung infection models with the five pathogens (and the specific strains) tested here are validated in a similar manner as neutropenic lung infection models with intranasal instillation of the inoculum. Thus, the model we propose here can be equally used to assess the efficacy of novel anti-infectives.

### Using aerosol delivery in acute pneumonia models

**Figure 3:**
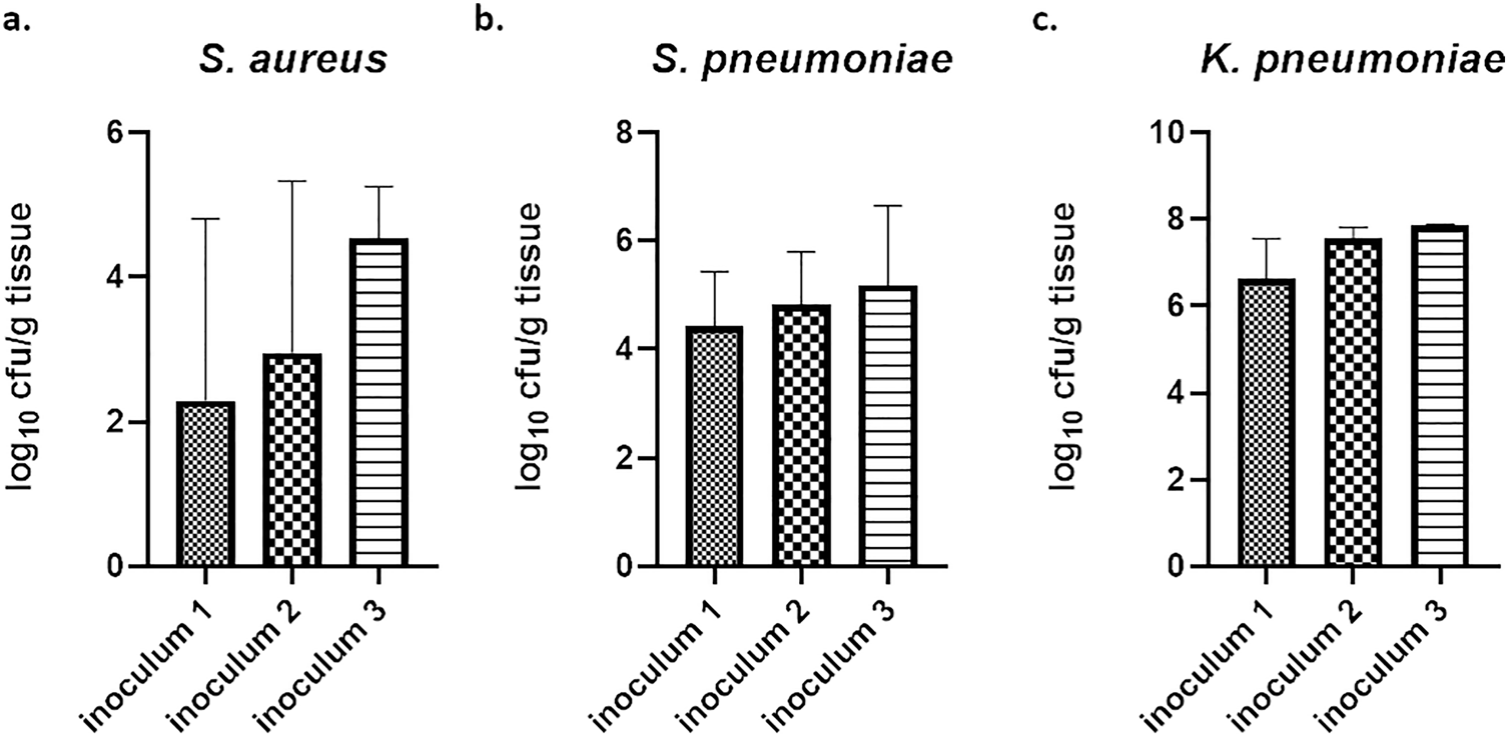
Bacterial burden after aerosol delivery of different ESKAPE pathogens in the acute pneumonia model during inoculum titration. Bacterial burden after 24 h after aerosolized delivery of three different inocula is shown for *S.* *aureus* (**a**), *S.* *pneumoniae* (**b**), *K.* *pneumoniae* (**c**) in the acute pneumonia model.

In a second step, we aimed to evaluate if a sustainable burden until 24 h was also achieved with nebulization when immunocompetent mice were used. Mice were infected in a similar manner as described for the neutropenic models using aerosolized delivery of the respective bacterial inoculum. First, different inocula were used (Table S1) to establish an infection. The infection with both Gram-positive strains of *S.* *aureus* and *S.* *pneumoniae* was established with mean log_10_ cfu/g tissue values of 4.3 and 4.6, respectively (Fig. S1b, Table S2). For both pathogens the highest inoculum dose (Table S2) was achieving the highest terminal bacterial burden with an acceptable standard deviation (Fig. 3a,b). However, no infection was established for the strains of *P.* *aeruginosa* and *A.* *baumannii* used in this study. Twenty-four hours post infection, bacteria were already cleared and no sustained bacterial burden was found in the lungs. Therefore, aerosolized delivery was not suited to induce an acute infection for *P.* *aeruginosa* and *A.* *baumannii*. By contrast, the infection with aerosolized delivery for the Gram-negative pathogen *K.* *pneumoniae* was successful and a good bacterial burden of around 7 log_10_ cfu/g tissue was detected 24 h post infection (Fig. S1b, Table S2). Moreover, with increasing inoculum dose an increased bacterial burden was observed (Fig. 3c).

**Figure 4:**
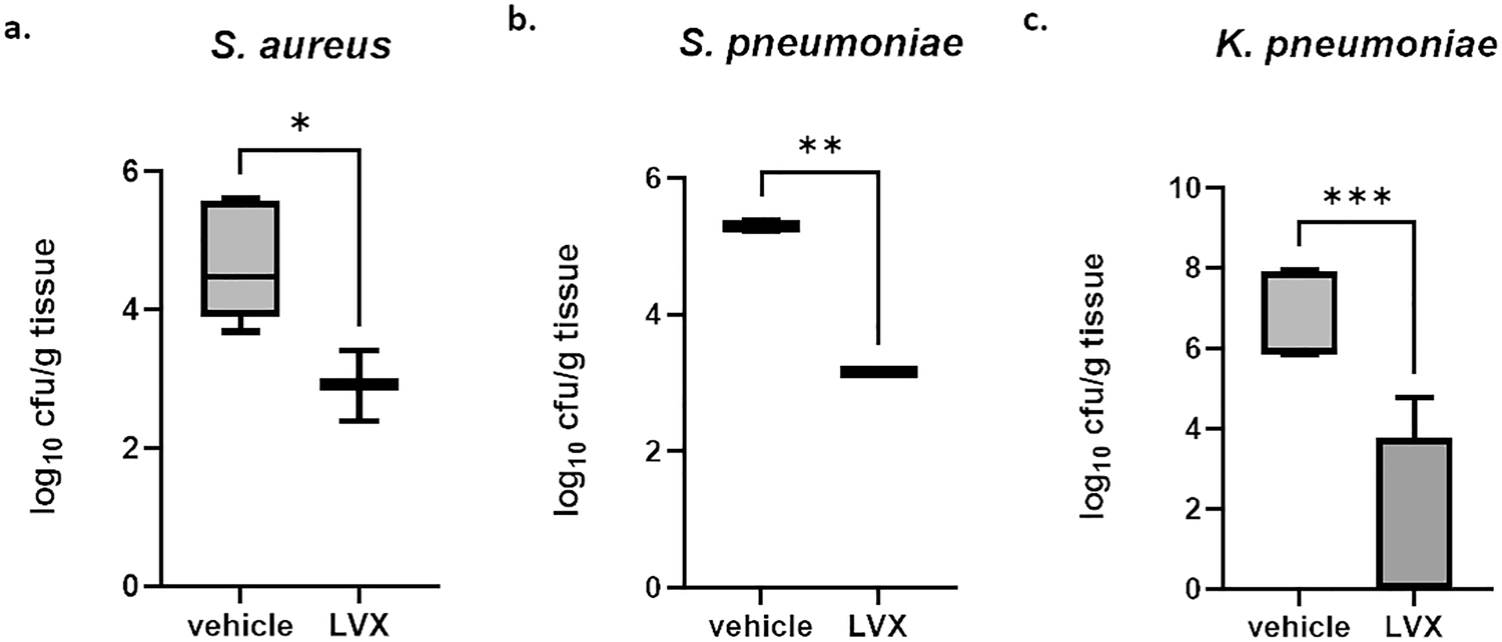
Validation of acute pneumonia models with levofloxacin. Bacterial burden after 24 h after aerosolized delivery is shown for *S.* *aureus* (**a**), *S.* *pneumoniae* (**b**), *K.* *pneumoniae* (**c**). Animals were treated with vehicle (left) or with levofloxacin (LVX, right). Levofloxacin did reduce bacterial burden at 24 h significantly for all pathogens tested. *p < 0.05, **p < 0.01, ***p < 0.001.

Similar to the neutropenic models, we aimed for a validation using the positive control antibacterial levofloxacin. Levofloxacin did reduce bacterial burden with *S.* *aureus* compared to the vehicle-treated group by 2 log_10_ units (Fig. 4a), which was a similar magnitude of reduction upon infection with *S.* *pneumoniae* (Fig. 4b). For *K.* *pneumoniae*, the mean reduction in bacterial burden in the levofloxacin group was around 4 log_10_ units compared to the vehicle-treated group (Fig. 4c).

In summary, the aerosolized delivery worked for three out of five pathogens tested. Moreover, we demonstrate using the positive control antibacterial levofloxacin as comparator that the model is entirely validated and usable to assess the efficacy of novel anti-infectives.

## Discussion

Neutropenic and immunocompetent, acute murine pneumonia models are established systems to provide a proof-of-concept as well as research primary pharmacology of novel antibacterials^13,16–22^. They help to understand PK/PD relationships and translate doses to obtain a good probability of target attainment in human clinical trials^23–25^. So far, the majority of these models predictive for efficacy in humans uses intranasal instillation of the pathogen^9,10^.

Herein, we show that aerosolized delivery of pathogens to induce pneumonia can be a viable alternative to conventional intranasal and intratracheal administration. In this study, we demonstrate that an infection was successfully established for five different strains in the neutropenic murine pneumonia model. Moreover, we showed that a high inoculum was needed to achieve a sustained bacterial burden for the five pathogens assessed. This was in line with previous reports of the inoculum size needed for murine pneumonia models^9^. Additionally, both *P.* *aeruginosa* and *A. baumannii* needed 0.01% mucin during inoculum preparation to achieve high terminal bacterial burden. In the context of an assessment of human-derived antibodies against *P.* *aeruginosa*, we have shown recently, that histopathological analysis showed an increased inflammation in the lungs in untreated animals^26^. This observation was in agreement with previous reports on histopathology results of murine pneumonia models^7,13,15^. It is known that mucin interacts with *P.* *aeruginosa* and that it attenuates its virulence and can impact its ability to form biofilms^27–29^. It has also been shown that mucin impacts the virulence of *A.* *baumannii*, in particular in the context of intraperitoneal infections^30,31^. Similarly, it has been demonstrated that mucin addition helps to establish intraperitoneal infections with Gram-negative pathogens, including *P.* *aeruginosa* and *A. baumannii*^32^. However, in that study mucin was not added during inoculum titration^32^. We hypothesize that low amount of mucin addition might have contributed to successful establishment of infection in the neutropenic models for *P. aeruginosa* and *A. baumannii*, whereas that was not possible in the acute lung infection models. Nevertheless, deciphering the exact mechanism of mucin addition during inoculum preparation was not within the scope of this study, but might be subject to further research. Additionally, it is known for *P.* *aeruginosa* as well as for *A.* *baumannii* that the establishment of acute lung infection models is challenging in general: For both, the size of the inoculum is critical^33,34^. If the inoculum is chosen too low, then rapid clearance of the infection in immunocompetent mice occurs as a result of the innate immune response in case of *P. aeruginosa*^34–36^. As a higher inoculum results in a more mucoid solution, a nebulization is not possible. Thus, aerosolized delivery has a limitation with regard to viscosity^37^. Therefore, in such cases intranasal or intratracheal administration of the pathogen is needed to enable an acute lung infection^10,38^. Nevertheless, aerosolized delivery of the pathogen was possible in case of three tested strains in this study. Finally, the models for all the pathogens tested in this study were validated with a positive control antibacterial, levofloxacin, demonstrating that they are suited to be used to also evaluate novel anti-infectives in a similar manner as models using intranasal instillation for infection^9^.

In general, aerosolized delivery of the inoculum has several advantages, which also contributes to a low standard deviation of terminal bacterial lung burden observed. One of these advantages is certainly that delivery is experimenter-independent because the flow rate and aerosol size is solely dependent on the nebulizer used^37^. The mesh nebulizer we used, i.e. Aeroneb® lab nebulizer, a vibrating mesh nebulizer, with the small nebulizer unit usually produces particle sizes between 2.5 and 4 µm (according to the manufacturer’s specifications). Moreover, nebulization was performed under controlled conditions, as required for animal experiments, in a temperature range of 21–22 °C and a relative humidity of 45–65%, despite the fact that the manufacturer also allows greater ranges for temperature and relative humidity. Choosing a nebulizer, which keeps the specifications (according to the manufacturer) at varying environmental conditions, renders the infection with aerosolized delivery more robust. As environmental conditions will be similar for murine pneumonia models worldwide because of animal welfare regulations, we did not change environmental conditions for the nebulization process. Furthermore, we used a small-sized nebulizer device making delivery of the inoculum simple without the need for extensive or huge special equipment. Additionally, intratracheal and intranasal administration as more invasive techniques might cause differences in the breathing rates, in case of intranasal administration as a result of obstruction of one nostril^39^. This might result in particle sizes not being defined, in contrast to a vibrating mesh nebulizer, causing higher standard deviation of the bacterial burden as observed by Bergamini and colleagues for intranasal and intratracheal administration^11^. We hypothesize that the particles generated by the nebulizer used in this study are much smaller than the ones generated through extensive breathing of the animal after intranasal administration. The particle size distribution generated by the vibrating mesh nebulizer might be more suitable for disposition in the respiratory tract as it has been shown that particles between < 5 µm deposit more easily in the respiratory tract, whereas those < 1 µm can be exhaled again^40,41^. By contrast, a lower standard deviation within groups is always advisable, as it will allow reducing group sizes with respect to statistical testing^42^. One might hypothesize that an evaporation process took place causing bacteria to be destroyed. However, for the Aeroneb® lab nebulizer used in this study, it has been observed, that smaller droplet sizes, below 2.5 µm, through evaporation were only achieved by varying experimental conditions extensively, such as by heating of the aerosol^43–46^. We chose the Aeroneb® lab nebulizer, because it is a well-characterized and specified system. It was out of the scope of this study to perform an in-depth characterization of the aerodynamic properties of the nebulization device going beyond the extensive characterizations already performed by the manufacturer. We did show that bacteria were growing in vivo starting at a burden of around 4 log_10_ units to a burden of 6–8 log_10_ units within 22 h. Thus, this suggested that bacteria were not destroyed by the nebulization process, but were still alive. A similar growth of bacteria was also seen in murine pneumonia models after intranasal instillation^10^. Finally, this contradicts the hypothesis that bacteria were destroyed by an evaporation process.

Thus, we believe that the low standard deviation we observe for the majority of pathogens tested in this study might be attributed to a homogenous particle size distribution of the bacterial inoculum resulting in low inter-animal variability. The low standard variation in combination with the device we used, only taking several seconds of aerosolization time, is the unique contribution compared to similar methods in literature. There is no need to involve large nebulization chambers and invest in highly specialized apparatus and training of personnel as described previously^13,47–49^. In the context of viral infections, nebulizers have already been used and were able to produce a sustained infection^50^. Moreover, in one study even the same Aeroneb® was used for assessment of a drug’s efficacy^51^. Nevertheless, the major part of studies needs higher volumes and longer time periods for inoculation. Bowling and colleagues have assessed differences between the former ‘gold standard’, the collison nebulizer, and the Aeroneb® lab nebulizer and determined less loss of pathogens during delivery in case of viral infections. With respect to initiation of bacterial inoculation, they were experiencing difficulties^52^. It has to be highlighted here that Bowling and colleagues used a chamber for inoculation. By contrast, we directly put the nebulizer over mouth and nose of the individual animal, which, presumably, causes less loss of the pathogen.

With the caveat that only one strain was used for the majority of the pathogens tested, sustained bacterial burden in lung tissue was obtained for the strains of the pathogens studied in the neutropenic pneumonia models as well as for the majority of strains in the acute pneumonia models. A good disposition of bacteria at the target site, i.e. lung, was observed at 2 h post infection. Furthermore, we were able to show that we get a similar terminal bacterial burden when using the same inoculum, but different strains of *A.* *baumannii*. This demonstrates that the method presented here is not restricted to specific strains, but has broader applicability, although inoculum titrations might be necessary for different strains to establish the model with aerosolized delivery.

Additionally, it is expected that due to the disposition of particles after aerosol delivery^14^, different analysis from the same sample material is enabled, such as RNA level determination of bacterial biomarkers beside cfu determination. It has been shown for the Aeroneb® we used in this study that a homogenous distribution of particle sizes and deposition into the lung is achieved^14,53–56^. Although it is mainly well documented that drug particles are well distributed across the lung when using an Aeroneb® device^56^, we argue that the same can be expected in a similar manner for bacterial deposition as it has already been shown in the context of respiratory syncytial virus (RSV)^50^. This gave us confidence that our assumption of a homogenous deposition of bacteria after aerosolized delivery is correct.

In summary, this small study showed that aerosol delivery for infection constitutes a suitable alternative for intranasal and intratracheal instillation. Mimicking the ‘natural route of infection’ this delivery method represents a refinement of the standard murine pneumonia models and encourages to consider this route for delivery, in particular when the lung tissue is needed for multiple readouts.

## Methods

### Bacterial strains

The following bacterial strains were used for in vivo studies: *S.* *aureus* ATCC 33591, *K.* *pneumoniae* ATCC 43816, *P.* *aeruginosa* ATCC 27853, *A.* *baumannii* ATCC 19606, *A. baumannii* NCTC 13301*, S. pneumoniae* ATCC 700905.

### Preparation of the inoculum for infection with *S. aureus* ATCC 33591 and *K. pneumoniae* ATCC 43816

Inoculi were prepared as described previously with the modification that 0.9% NaCl-solution was used instead of PBS^57^. For the neutropenic and the acute lung infection model an inoculum of 2 × 10^9^ cfu/ml was used for *S.* *aureus* ATCC 33591 as well as for *K.* *pneumoniae* ATCC 43816.

### Preparation of the inoculum for infection with *P. aeruginosa* ATCC 27853

The inoculum was prepared as follows: on day -1 the *P.* *aeruginosa* strain was streaked out onto a blood agar plate and incubated at 37 °C. Then one single colony was inoculated into LB medium (diluted 1:6 in water) containing 0.01% mucin and incubated at 120 rpm and 37 °C. On day 0 bacteria were centrifuged for 15 min at 4,000 rpm and washed twice in 0.9% NaCl-solution. Then they were adjusted to an OD of 10. For infection with *P.* *aeruginosa* an inoculum of 5 × 10^9^ cfu/ml was used.

### Preparation of the inoculum for infection with *A. baumannii* ATCC 19606 and NCTC 13301

The respective strain was streaked out from a glycerol culture onto a blood agar plate and incubated at 37 °C overnight. A few colonies were inoculated in a mixture of MHB diluted in water (one part MHB and five parts water) with 0.01% mucin and incubated for 14–15 h at 120 rpm and 37 °C. The following day, the culture was centrifuged at 4000 rpm and 4 °C for 15 min when it reached an OD600 between 0.5 and 0.6. Then it was washed twice with 0.9% NaCl-solution and centrifuged for 10 min at 4000 rpm and 4 °C. Finally, the pellet was resuspended in 0.9% NaCl-solution and the OD600 was adjusted to 10. For infection with *A.* *baumannii* an inoculum of 2 × 10^9^ cfu/ml was used.

### Preparation of the inoculum for infection with *S. pneumoniae* ATCC 700905

The strain was streaked out from a glycerol culture onto a blood agar plate and incubated at 37 °C overnight. A few colonies were inoculated in THY medium and incubated overnight at 120 rpm and 37 °C. The following day the overnight culture is diluted 1:100 in THY incubated at 120 rpm and 37 °C until it reached an OD600 of around 0.5–0.6. Then it was washed twice with 0.9% NaCl-solution and centrifuged for 10 min at 4000 rpm and 4 °C. Finally, the pellet was resuspended in 0.9% NaCl-solution and the OD600 was adjusted to 5. For infection with *S.* *pneumoniae* an inoculum of 3 × 10^9^ cfu/ml was used.

### Mice

The animal studies were conducted in accordance with the recommendations of the European Community (Directive 2010/63/EU, 1st January 2013). All animal procedures were performed in strict accordance with the German regulations of the Society for Laboratory Animal Science (GV- SOLAS) and the European Health Law of the Federation of Laboratory Animal Science Associations (FELASA). Animals were excluded from further analysis if sacrifice was necessary according to the humane endpoints established by the ethical board. All experiments were approved by the ethical board of the Niedersächsisches Landesamt für Verbraucherschutz und Lebensmittelsicherheit, Oldenburg, Germany. Animals were kept in individually ventilated cages with a 10h/14h dark/light cycle and had access to food and water ad libitum. The animals were housed in individually ventilated cages in an animal facility with a room temperature of 21–22 °C and a relative humidity in the range of 45–65%. The study is reported in accordance with the ARRIVE guidelines.

### Neutropenic and acute pneumonia models

#### General

Female, eight weeks-old, CD-1 mice (Charles River, Germany) were used for both models (n = 6 animals per study per strain for the 24 h-time point and n = 2 animals for the 2 h-time point). In case of the neutropenic pneumonia model, animals were rendered neutropenic by administration of 150 mg/kg and 100 mg/kg cyclophosphamide intraperitoneally on day-4 and-1, respectively. On the day of infection (day 0), mice received 15 µl (neutropenic models) or 50 µl (acute models) of the respective strain administered via an Aeroneb® nebulizer device (Kent Scientific) under anesthesia with 100 mg/kg ketamine and 10 mg/kg xylazine (both administered intraperitoneally). Nebulization procedures were performed within a BSL-2 safety cabinet in an animal facility with a room temperature of 21–22 °C and a relative humidity of 45–65%. Two hours (in case of the neutropenic models) and 24 h after infection (neutropenic and acute models), mice were euthanized, blood was removed from the heart and lungs were aseptically removed. Whole blood was collected into Eppendorf tubes coated with 0.5 M EDTA and immediately spun down at 13.000 rpm for 10 min at 4 °C. The plasma was transferred into a new Eppendorf tube and then stored at − 80 °C until analysis. Organs were homogenized in 0.9% NaCl-solution and plated onto agar plates in duplicates in serial dilutions and incubated at 37 °C for 24 h.

### Inoculum titration for model development

To establish the inoculum for both the neutropenic and acute lung infection models, three inocula were tested. The inocula were prepared as described in the section for preparation of the inoculum for the respective strain. For the inoculum titration 1/5, 1/2 and the inoculum size as described in the section ‘preparation of the inoculum’ were used, corresponding to ‘inoculum 1’ for 1/5 of the original inoculum, to ‘inoculum 2’ for the 1/2 of the original inoculum and ‘inoculum 3’ for the original inoculum (Table S1). In case of *A. baumannii* strain ATCC 19606 as well as for *P.* *aeruginosa*, the first tests were performed without addition of mucin during inoculum preparation.

### Assessment of bacterial growth over time and validation of the model with the positive control

Levofloxacin 100 mg/kg IP served as a positive control antibacterial. The animal dose in mice of 100 mg/kg corresponds to a human equivalent dose of 8.1 mg/kg^58^. For *S. aureus*, *A. baumannii* ATCC 19606 and *P.* *aeruginosa*, it was administered once daily at t = 2 h. For *K.* *pneumoniae* and *S.* *pneumoniae* it was administered three times daily at t = 2, 6 and 10 h. Levofloxacin was not used for the *A.* *baumannii* strain NCTC 13301 as levofloxacin was not sensitive enough according to literature^59^. Moreover, bacterial burden in lung tissue for the neutropenic models was assessed at time points t = 2, 5 and 24 h, whereas bacterial burden in the acute models was assessed at time points t = 24 h.

### Statistical testing

Statistical testing was performed using GraphPad Prism 9.5.1 software. For the acute infection models a two-tailed unpaired t-test was performed. For the neutropenic infection models a mixed-effect analysis with Fisher’s LSD test was performed for determination of significance at t = 24 h between the vehicle and the levofloxacin group.

### Supplementary Information

Supplementary Information 1.

Supplementary Video 1.

Supplementary Video 2.

Supplementary Video 3.

## Data Availability

The authors confirm that the data supporting the findings of this study are available within the article.
