# Supplementary material for: Aerosolized delivery of ESKAPE pathogens for murine pneumonia models
Source: Sci Rep. 2024 Jan 31;14:2558. doi: 10.1038/s41598-024-52958-9 (PMC10830452; doi:10.1038/s41598-024-52958-9)
Supplement: Supplementary file 1 — Supplementary Information 1. [file 41598_2024_52958_MOESM1_ESM.pdf]

Supplemental Information

Aerosolized delivery of ESKAPE pathogens for murine pneumonia models

Rox, K[1,2]*, Medina, E[2,3]

[1]Department of Chemical Biology, Helmholtz Centre for Infection Research (HZI), Inhoffenstraße 7, D-38124 Braunschweig, Germany

[2]German Center for Infection Research (DZIF), Partner site Hannover-Braunschweig, D-38124 Braunschweig, Germany

[3]Infection Immunology Group, Helmholtz Centre for Infection Research (HZI), Inhoffenstraße 7, D-38124 Braunschweig, Germany

Correspondence: Dr. Katharina Rox, e-mail: katharina.rox@helmholtz-hzi.de , Phone: +49 531 6181 4409

Table of content

Supplemental Figure 3

Figure S1. Bacterial burden after aerosol delivery of different ESKAPE pathogens in the standard neutropenic and acute pneumonia model. 3

Supplemental Tables 3

Table S1. Inocula used for aerosolized delivery for inoculum titration (expressed as cfu/ml). 3

Table S2. Bacterial burden after 24 hours in lung tissue expressed as mean and standard deviation in cfu/g tissue in the neutropenic and the acute pneumonia model. 3

Supplemental video files description 4

Video file S1 4

Video file S2 4

Video file S3 4

Supplemental Figure

Figure S1. Bacterial burden after aerosol delivery of different ESKAPE pathogens in the standard neutropenic and acute pneumonia model.

Nebulized delivery of pathogens results in a mean bacterial burden of 5-7 log10 cfu/g tissue 24 hours after infection in the neutropenic pneumonia model (a). A bacterial burden of around 4 log10 cfu/g tissue is found for S. pneumoniae and S. aureus, whereas K. pneumoniae showed a bacterial burden of around 6 log10 cfu/g tissue (b).

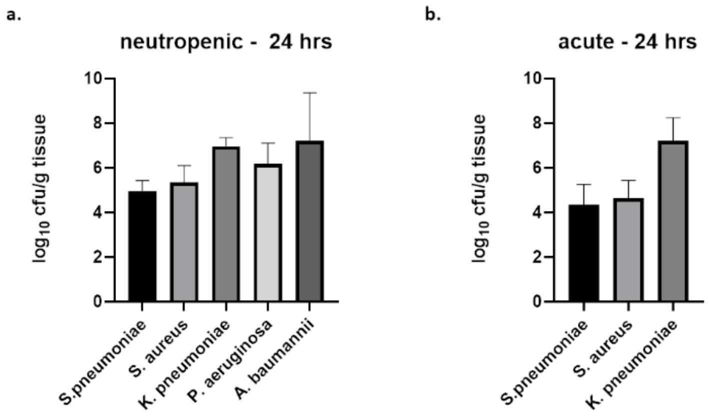

Supplemental Tables

Table S1. Inocula used for aerosolized delivery for inoculum titration (expressed as cfu/ml).

	S. aureus	*S. pneumoniae*	*K. pneumoniae*	*P.aeruginosa*	*A. baumannii ATCC*
inoculum 1	$4x10^8$	$6x10^8$	$4x10^8$	$1x10^9$	$4x10^8$
inoculum 2	$1x10^9$	$1.5x10^9$	$1x10^9$	$2.5x10^9$	$1x10^9$
inoculum 3	$2x10^9$	$3x10^9$	$2x10^9$	$5x10^9$	$2x10^9$

Table S2. Bacterial burden after 24 hours in lung tissue expressed as mean and standard deviation in cfu/g tissue in the neutropenic and the acute pneumonia model.

	Neutropenic pneumonia model [log_{10} cfu/g tissue]	*Acute pneumonia model [log_{10} cfu/g tissue]*
S. pneumoniae	5.0 ± 0.5	4.3 ± 0.9
S. aureus	5.3 ± 0.8	4.6 ± 0.8
K. pneumoniae	7.0 ± 0.4	7.2 ± 1.0
P. aeruginosa	6.2 ± 0.9	-
A. baumannii	7.2 ± 2.1	-

Supplemental video files description

Video file S1

The video file shows the assembly of the Aeroneb® lab nebulizer.

Video file S2

The video file shows how to add the inoculum (in the video PBS) to initiate the nebulization.

Video file S3

The video shows how to disassemble and remove residual droplets to assure reproducible performance of the device.
